# Biofilm through the Looking Glass: A Microbial Food Safety Perspective

**DOI:** 10.3390/pathogens11030346

**Published:** 2022-03-12

**Authors:** Sapna Chitlapilly Dass, Rong Wang

**Affiliations:** 1 Department of Animal Science, Texas A&M University, College Station, TX 77845, USA; 2 Roman L. Hruska U.S. Meat Animal Research Center, Agricultural Research Service, U.S. Department of Agriculture, Clay Center, NE 68933, USA; rong.wang@usda.gov

**Keywords:** biofilm, microbial ecology, black queen hypothesis, adaptive response

## Abstract

Food-processing facilities harbor a wide diversity of microorganisms that persist and interact in multispecies biofilms, which could provide an ecological niche for pathogens to better colonize and gain tolerance against sanitization. Biofilm formation by foodborne pathogens is a serious threat to food safety and public health. Biofilms are formed in an environment through synergistic interactions within the microbial community through mutual adaptive response to their long-term coexistence. Mixed-species biofilms are more tolerant to sanitizers than single-species biofilms or their planktonic equivalents. Hence, there is a need to explore how multispecies biofilms help in protecting the foodborne pathogen from common sanitizers and disseminate biofilm cells from hotspots and contaminate food products. This knowledge will help in designing microbial interventions to mitigate foodborne pathogens in the processing environment. As the global need for safe, high-quality, and nutritious food increases, it is vital to study foodborne pathogen behavior and engineer new interventions that safeguard food from contamination with pathogens. This review focuses on the potential food safety issues associated with biofilms in the food-processing environment.

## 1. Introduction

Our understanding of the microbial world has evolved from single-species existence to highly complex and diverse microbial communities [1,2]. Previously, microbiological studies have involved studying microorganisms in axenic stetting, overlooking the fact that in many environments, microorganisms coexist [3]. With the recent shift in studying microorganisms as a mixed community, there has been a surge of research focusing on biofilms and cell–cell communication, thus sparking the importance of examining multispecies systems and their combined metabolic properties [4,5,6,7,8]. Biofilms are complex communities that are anchored to a substratum and are enveloped in an extracellular polymeric substance (EPS) matrix [9,10]. The EPS layer in the biofilm provides higher resilience to various environmental stress and resistance to antimicrobial, chemical, or sanitizer treatments [11,12]. Biofilm formation is highly variable among different microorganisms, thus adding complexity in understanding the mechanism of biofilm formation [10,13,14]. Biofilms are of high medical and economical significance, as they have been associated with chronic illness, food contamination, antibiotic tolerance, plant health, bioremediation, natural product discovery, and waste-water treatment [1,3,10,11,15,16].

In food-processing environments, biofilms have been a major cause for food spoilage associated economic losses and food safety issues leading to number of outbreaks [16,17,18,19]. An estimated 9.4 million foodborne diseases due to known pathogens are reported each year in the United States [20,21,22]. In the food industry, owing to high risk of food safety and spoilage involving the persistence of biofilm, research has been directed towards better understating of biofilm formation, interventions, and approaches to mitigate them.

The aim of this review is to discuss the potential food safety issues associated with biofilms in the food-processing environment.

## 2. Mechanisms for Biofilm Formation: Attachment, Maturation, and Dispersion of Microorganisms to the Food-Processing Environment Surface

Formation of biofilms is a dynamic process and involves numerous steps that run in tandem to form a highly structured community of microbial cells, which are enveloped within a protective outer covering. The persistence of biofilms in a food-processing environment is closely associated with the response to several abiotic and biotic factors [12,18,23]. The biotic factors that are vital in biofilm formation are type of microorganism, cell–cell communications, metabolic activity, growth phase, interactions with other microorganisms, and gene regulation [10,20,24]. The abiotic factors that are fundamental in the formation of biofilm in a food-processing environment are temperature, surface properties, nutrients, pH, water activity, and stressing agents (disinfectants and antimicrobial agents) [9,18,19]. How microorganisms accomplish this complex intricate mechanism in the formation of biofilms in food-processing facilities is explained below.

### 2.1. Attachment of Biofilm to the Substratum

Attachment of microorganism to a surface can be active or passive depending on their solid–liquid interface between the surface and an aqueous medium [9], cell surface properties of the microorganism [23], and motility of the microorganism or gravitational transportation of their planktonic state [16]. Physical forces associated with bacterial adhesion are collectively known as the DVLO (Derjaguin, Verwey, Landau, and Overbeek) forces [24]. The DLVO theory [25,26] has been used to describe the net interaction between a cell and substratum as a balance between two additive factors, resulting from attractive and repulsive interactions from the overlap between the cell and the substratum [26]. An extended DVLO theory takes into consideration hydrophobic/hydrophilic and osmotic interactions [27] and has also been described in terms of thermodynamic interaction [28].

The first stage in biofilm formation is attachment of the microorganisms to the substratum; attachment is a two-step process: first is reversible attachment followed by irreversible attachment. The initial attachment of the microorganisms is reversible attachment, as the microorganisms that initiated the process of biofilm formation are not yet committed to the cell differentiation process that leads to the biofilm formation [12,23]. In the food-processing facility, any surface is prone to formation of biofilm, such as stainless steel, wood, glass, plastic, and also the food product itself. The surface properties, such as texture of the surface—rough or smooth—, hydrophobicity, pH, nutrients, water activity, and stressing agents, impact the attachment [17]. The biofilm attachment is increased on rough surfaces when compared to smooth surfaces, and this is due to the decrease shear forces on rough surfaces [9]. Stainless steel is one of the most used surfaces in food-processing facility, as it is resistant to corrosion from cleaning agents and has high durability [29,30]. Due to regular wear and tear, stainless steel surfaces develop cracks, and this encourages the lodgment of organic materials in these cracks and crevices, thus attracting microorganisms to gather [18]. These is an ideal habitat for microbial life for biofilm formation, as these are hard-to-clean areas, and due to the continuous functioning of the processing facility, deposition of organic material will be constant.

Cell surface property, such as hydrophobic and hydrophilic property, also plays a role in bacterial attachment [19]. The microbial cell surfaces that are hydrophobic tend to attach to surfaces with hydrophobic surfaces, while hydrophilic microbial cell surfaces attach to hydrophilic surfaces [12]. Research has shown that attachment of microorganisms to surfaces that are hydrophobic, such as plastic and Teflon, are stronger when compared to glass or metals [12,30,31,32,33]. For example, *Listeria* and *Salmonella* form stronger biofilms on hydrophobic surfaces than the hydrophilic surfaces [32,34]. Other than processing facility surfaces, biofilms are formed on the incoming food materials [16].

### 2.2. Maturation of Biofilm

**Figure 1:**
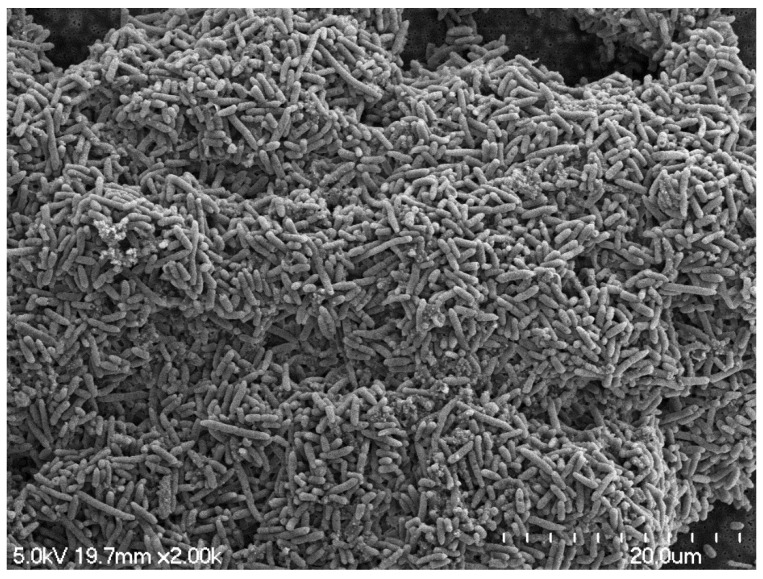
SEM image of the multilayer drain biofilm formation on a stainless steel chip, grown at 7 °C for five days under static conditions.

Depending on the nutrient source for the biofilm, the biofilm shapes into a mushroom (Figure 1) or flat shape [17]. The maturation can vary and can happen from 5 to 10 days [33]. During maturation, gene alteration takes place in genes encoding for translation, metabolism, membrane transport, and/or secretion and gene regulation [23].

One of the regulators for maturation of biofilm is quorum sensing [35,36]. Quorum sensing is a cell–cell communication strategy [36]. The mechanism of cell–cell communication is explained in the later part of the review. The matured biofilm can be composed of multi-layers of micro-colonies or a monolayer of cells [10]. The monolayer or thin layer of biofilm is due to the lack of las quorum sensing system, which initiates the biofilm formation, and the thickness of the biofilm is determined by levels of las quorum sensing system [35,36]. The complex architecture of the mature biofilm is actively maintained by rhamnolipid surfactants [37], produced by cells within the biofilm, maintaining the open spaces or channels surrounding macrocolonies by inhibiting colonization by invading planktonic cells [36,37,38]. Figure 1 illustrates the multilayer drain biofilm formation on a stainless steel chip, grown at 7 °C for five days under static conditions. Multispecies biofilms contain specific subsets of species derived from the planktonic phase [38].

### 2.3. Dispersion

**Figure 2:**
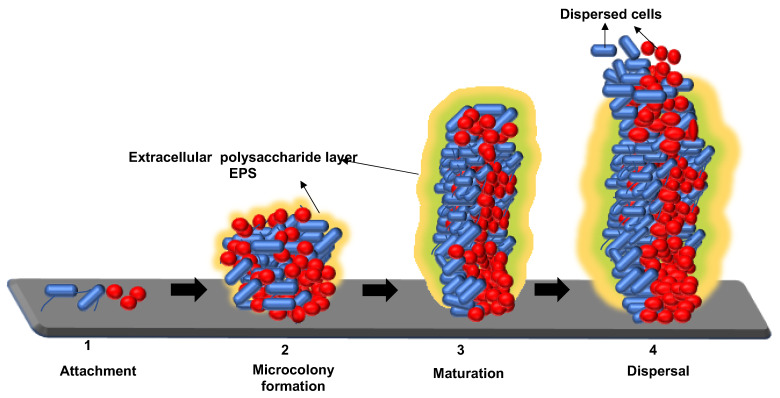
Illustration of different stages of biofilm formation: (**1**) Attachment, (**2**) microcolony formation, (**3**) maturation, and (**4**) dispersion.

The last step in the biofilm formation is the dispersion of the cells, which allows the cells to revert back to its planktonic state [39]. The stages of biofilm formation and dispersion of cell are illustrated in Figure 2. Bacterial cells in the biofilm adapt to their changing environment by adjusting their Bis-(3′-5′)-cyclic dimeric GMP (c-di-GMP), which is an essential secondary messenger the bacteria employ in the control of biofilm formation and dispersal [39]. When the biofilm is exposed to adverse stress conditions and starvation, the bacterial cells reduce their c-di-GMP and activate phosphodiesterase, resulting in dispersal of biofilms [9,12,39]. Detachment is an active process, which allows for the colonization of new niches and also starvation, which is considered as a reason of detachment and allows bacteria to search for a nutrient-rich environment [33,39]. The transition period in which a dispersed cell reverts back to planktonic state is critical, as it can be highly virulent before finding a new niche [39].

Biofilm harborage in food-processing facilities, regardless of the food that is being processed, has shared common areas that encourage the growth of biofilm. The areas of a food-processing environment where biofilms are formed include floors, drains, water distribution pipes, and difficult-to-clean surfaces, such as the back of the conveyor belts coils on spiral freezers at frozen food plants [40]. These surfaces become hotspots that attract biofilm development due to poor accessibility and difficulty for regular maintenance of hygiene and sanitation maintenance [41].

The broiler slaughter industry generates residue rich in protein and lipids, which are deposited on surfaces [42], such as stainless steel, aluminum, nylon, rubber, plastic, polystyrene, and glass [42,43]. This favors the formation of biofilms becoming a potential source of contamination that can be transferred to poultry products or to their packaging, becoming a constant threat of recontamination [44]. Major pathogen contaminants of poultry products are *Salmonella* and *Campylobacter*, which represents high-risk foodborne infections to human and have been isolated from poultry environmental biofilms [42,43,44,45].

Ready-to-eat food (RTE) is well-processed food that requires no further processing before consumption [46]. These are considered very high-risk foods, as they are consumed without undergoing any bactericidal activity [47]. RTE foods could potentially be susceptible to cross-contamination during processing or post-processing [16,47]. Food-processing facility environmental biofilm has been considered to a source of contamination, specifically for delicatessen meat, an RTE food product, with *E. coli*, *Salmonella* spp., *Campylobacter* spp., and *Listeria monocytogenes* [47,48]. Presence of *Vibrio cholerae*, *Pseudomonas* spp., *Bacillus* sp., and *L. monocytogenes* in salmon and environmental biofilms in cold-smoked salmon processing facilities has been a major food safety concern [49,50,51,52,53].

The bacterial contamination of fresh produce is a major issue in food-processing environments and increases the risk of foodborne illness [54]. Fresh-produce-related outbreaks have been reported in multiple states in the past couple of years [55,56,57]. Studies show that the attachment and internalization of pathogenic enteric bacteria on plants have caused many disease outbreaks due to consumption of fresh produce [54]. Pre-and post-processing of vegetables may lead to the transmission of pathogenic biofilms to food-contact surfaces and can cause human health and food safety issues [54,55,56,57,58]. In fresh produce processing, in addition to environmental biofilm harborage, interactions between human pathogens and plant tissues have to be considered [59]. Human pathogens, such as *E. coli* O157:H7 [60,61,62], *L. monocytogenes* [59], and *S. enterica* [62,63,64], have been found to be capable of attaching to and colonizing the surfaces of growing plants. Once attached, human pathogens can form biofilms on plant tissues [59]. The formation of biofilm within the plant tissue could be attributed to the main failure of washing treatments of fresh produce to remove or inactivate human pathogens on produce surfaces [54,59].

In the meat industry, Shiga-toxin producing *Escherichia coli* (STEC) O157:H7 is the most commonly identified STEC serotype that causes foodborne outbreaks and multiple clinical diseases, including bloody diarrhea and HUS. Cattle are a major reservoir for this pathogen, and the harborage of STEC O157:H7 on animal hides has been identified as the major source of meat carcass contamination during slaughtering and processing [65,66]. However, the presence of STEC O157:H7 on meat-processing equipment, such as conveyor belts [67] and other food-contact surfaces [68,69], has been detected, and the biofilm-forming ability of this pathogen under various conditions is well related to its persistence in the meat-processing environment. More importantly, a wide variety of materials commonly used in the meat industry may become hosts of STEC biofilms, such as stainless steel, aluminum, nylon, Teflon, rubber, plastic, glass, polyvinyl chloride, and polyurethane, etc. For instance, STEC O157:H7 was able to attach and form biofilms on high-density polyethylene and stainless steel surfaces within a wide temperature range (4 °C–15 °C), and the different types of surface materials would affect bacterial biofilm-forming potency. Available studies more relevant to the meat industry also suggest that the strong biofilm-forming ability by certain STEC O157:H7 and *Salmonella enterica* strains as well as the resultant high sanitizer tolerance might play important roles in meat contamination incidence at commercial plants, such as the “High Event Period” (HEP) beef trim contamination by STEC O157:H7 [70,71,72].

The transition from reversible to irreversible attachment happens when the weak interaction of the bacteria is transformed to strong permanent bond with favorable conditions and enveloped in an EPS layer [33,73]. The EPS layer are biosynthetic polymers that can be highly diverse in chemical composition and may include substituted and unsubstituted polysaccharides, substituted and unsubstituted proteins, nucleic acids, and phospholipids [5]. EPS is highly hydrated, as it can incorporate large amounts of water into its structure by hydrogen bonding [9,32]. Because EPS is highly hydrated, it prevents desiccation in some natural biofilms [34]. EPS may also contribute to the antimicrobial resistance properties of biofilms by mass transport of antibiotics through the biofilm [9,19,74]. They are resilient structures and resistant to surfactants, sanitizers, detergents, or use of high temperature [23]. Removal of biofilms can require combinations of strong shear mechanical force, high temperatures, detergents, and sanitizers [75,76,77].

## 3. Architecture of Biofilm

Biofilms can be composed of either a single species or a community derived from multiple microbial species. Biofilm formation capacity varies between different microorganism, and it is influenced by factors such that one type of bacteria can be a strong biofilm producer under a certain environment and become weak in another environment [37]. There are various factors that determine the structure of the biofilm, such as surface and interface properties, nutrient availability, the composition of the microbial community, and hydrodynamics [78].

Biofilms in either single-species or multispecies consortia exhibit similar overall structural features [9]. The biofilm is initiated with the coaggregation with genetically distinct bacteria attach to one another via adhesin and receptors present on the cell surface [79,80,81]. Coaggregation of cells to form biofilms has two possible routes: Route one is by single cells in planktonic state specifically recognizing and adhering to genetically distinct cells in the developing biofilm [38]. The second route is by the prior coaggregation in planktonic cells of secondary colonizers followed by the subsequent adhesion of this co-aggregate to the developing biofilm [38,82]. In both cases, planktonic cells adhere to cells in the biofilm in a process known as co-adhesion [38]; thus, the co-adhered cells become part of the biofilm community [38,83].

**Figure 3:**
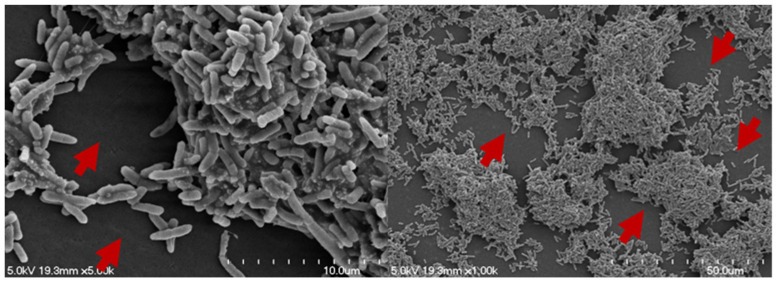
SEM image of the interstitial voids in a mixed species drain biofilm, developed on a stainless steel chip at 7 °C for five days under static conditions. The red arrows point to the interstitial voids.

Biofilms are separated by interstitial voids (fluid channels), and the whole structure is encased in an EPS layer [12]. The interstitial voids form an integral part of the biofilm structure [84]. Researchers have been able to demonstrate that water flow through these interstitial voids [37,84]. Figure 3 illustrates the interstitial voids in a mixed-species drain biofilm, developed on a stainless steel chip at 7 °C for five days under static conditions, including the interstitial voids, transports nutrients, oxygen, metabolites, waste, and antibiotics [37,73].

The levels of oxygen distribution varied in the different layers of the biofilm, with gradients of oxygen depletion towards the deeper parts of biofilms [83,84]. Researchers found that anoxic areas exist within biofilms and that oxygen depletion extends beyond the area of bacterial cells [84,85]. Oxygen-consuming bacteria through respiration may be attributing to the anoxic regions resulting in rapid reduction in the oxygen concentration from the surface to the interior of the microcolonies, and genes associated with anaerobic respiration are induced in the interior of microcolonies [78,85]. The oxygen concentration in the biofilm decreases with increasing depth. Similarly, the concentration of any nutrient that is consumed in the biofilm will also decrease with depth into the biofilm and distance from the nutrient source [86,87,88]. In addition, the characteristics of the nutrient accumulation inside biofilm of natural microbial consortia changes in synchrony with the surrounding nutrient sources [89].

## 4. Interactions of Microorganism in a Biofilm Community

Survival of an organism in the environment depends on its ability to sense and respond to the changes in its surroundings. Cell-to-cell communication is key for social construct of biofilm formed by different groups of microorganisms and to escape adverse environmental conditions [1,23,88]. These communications are essential for cell attachment, maturation, and detachment from biofilms [90]. The signals produced by microorganisms vary among different groups [1]. In a multispecies biofilm, how these communication signals are expressed and interpreted by other species are important in establishing a successful biofilm.

### 4.1. Signaling in Interspecies, Intraspecies, and Cross-Kingdom Communication

Signaling molecules are often referred to as autoinducers; these are small, diffusible molecules [88]. A number of microorganisms use diffusible signal molecules to monitor the population density and to modulate their behavior in response to their environment [90]. At low concentrations, some antibiotics also act as signaling molecules, and this may contribute to the antibiotic tolerance [90]. Cell–cell communication allows groups of microorganisms to behave in a coordinated fashion to regulate biofilm formation [91,92].

Gram-positive and -negative bacteria produce and sense small diffusible compounds called autoinducers in cell-to-cell communication. The mechanisms for producing and detecting autoinducers is referred to as quorum sensing [91]. As the name suggests, quorum sensing provides demographic information about the population level to other microbial groups nearby that senses the autoinducer. Other than population sensing, these diffusing autoinducers also provide information on spatial distribution of cells and conditions of the local environment [91]. The production of autoinducers is directly proportional to cell density, when the cell population increases, which in turn produces more of the autoinducer molecules [13,14,88,91].

In gram-positive bacteria, the cell signaling system is composed of two component systems: a membrane bound sensor kinase and cytoplasmic transcription factors [11]. Gram-negative bacteria are composed on LUX IR circuit [93]. Species–species communication allows recognition of self in a mixed population and also a mechanism to sense other bacteria [11]. Gram-positive and gram-negative bacteria communicate with by producing a signal molecule AI-2, which is common in both [16]. The signal molecule facilitates interspecies communication to sense each other’s presence and enable the biofilm formation.

Cross-kingdom cell signaling occurs between both prokaryotes and eukaryotes. The signaling involves small molecules, such as hormones by eukaryotes and hormones similar to chemicals produced by bacteria [13,94]. The cross-kingdom communication helps in establishing biofilms in animal and plant tissues. In food-processing facilities, the food matrix can be of both plant and animal origin and introduction of biofilms through their tissues can be a high food safety risk [12,13,93,95].

### 4.2. Metabolic Interactions

Microbial communities in the biofilm possess a combined metabolic activity or obligatory mutualistic metabolism shared among all the species [37]. Metabolic interactions among microorganisms leads to the success of the biofilm [96]. These interactions contribute to division of labor among the different groups and lead to an increased virulence [11]. Combined metabolic activity of microorganisms enable a microbial community to survive with minimal energy resources [11]. Successful establishment of multispecies biofilm can result from an association between metabolically cooperative organisms that facilitates interspecies substrate exchange and the removal or distribution of metabolic products [91,93]. Biofilms provide an ideal environment for the establishment of obligatory mutualistic metabolism or syntrophic relationships [10]. Syntrophism is a special case of symbiosis in which two metabolically distinct types of bacteria depend on each other to utilize certain substrates, typically for energy production [10,93].

### 4.3. Enhanced Biofilm Formation through Microbial Interactions

Synergistic interactions can enhance the biofilm formation. In a study carried out by Bharathi et al., 2011 [93], when four poor biofilm formers were cocultured, it enhanced the potential to form strong multispecies biofilms [97]. Metabolic interactions that enhanced coaggregations and organized spatial distribution could be responsible for the shift from weak biofilm formation to strong biofilm formers [2,98,99]. Species that do not form biofilms as single strains may benefit from the advantages associated with biofilm formation, including enhanced protection from external stress and expanded niche availability, through engagement with multispecies communities [78,99].

Enhanced biofilm formation and protection to less tolerant species with low ability to form biofilms has been observed in multispecies biofilms [78,91,100]. This is achieved by cooperative behavior between bacterial communities rather than competition among them. With the higher the number of species, the complexity of interactions increases. The cooperation in a multispecies biofilm is explained in the “Black Queen Hypothesis” [101]. This hypothesis considers cooperation in complex bacterial communities as being a consequence of species adapting to the presence of each other [11,78]. Some species in the complex community delete vital function or pathways that are provided by the surrounding bacteria, leading to communal dependency [2,88] and thus leading to irreversible commitment to coexistence [102].

## 5. Molecular Basis of Sanitizer Tolerance

Overall, the molecular mechanism of sanitizer tolerance has not been well characterized. Unlike bacterial antibiotic resistance, which is often based on one or more target gene mutations against the drug’s selective toxicity, most of the disinfectants/sanitizer reagents are complexes of antimicrobials inactivating multiple cellular components rather than acting on a single and specific cell target; thus, one single mutation usually would not be able to confer sufficient tolerance. However, bacterial tolerance to certain disinfectants, such as triclosan, as a result of mutations altering bacterial target and specific cellular components was also reported [102]. In addition, increased tolerance of *Salmonella* strains to oxidizing biocides was found to be associated with the production of neutralizing enzymes and DNA-repairing enzymes [103,104].

Non-specific alteration of bacterial cell envelop and cell wall structures that impact cell permeability has been deemed as the common mechanism associated with sanitizer tolerance [105]. Up-regulation of efflux pump activity that extrudes toxic substances, such as sanitizers and antimicrobials, from the cells is another critical strategy. Sanitizer tolerance associated with plasmids and integrons carrying efflux-related transporters, e.g., qacE, was investigated [106]. Up-regulation of efflux pump-encoded genes Mar, Sox, and AcrAB-TolC was observed in *E. coli* biofilm cells being treated with multiple antimicrobial agents [72].

Biofilms by *E. coli* O157:H7 strains isolated from HEP beef contamination exhibited strong tolerance to common sanitizers, which was not solely dependent upon biofilm mass development or bacterial EPS expression [68] but also positively correlated to the strains’ high pO157 plasmid copy number [107], suggesting such high tolerance might be related to the regulation mechanisms influenced by the presence of the plasmid. Even though the role of the pO157 plasmid, that is, highly conserved in *E. coli* O157:H7 strains, in bacterial survival capability under stressful and adverse circumstances has not been well characterized, this plasmid was found encoding critical virulence factors required for bacterial optimal survival and persistence in the environment and in the host [108,109,110]. The effects of the pO157 plasmid on bacterial colonization and survival capability might take place either directly through increased gene expression on the pO157 plasmid or via regulation of chromosomal gene expressions by the presence of the plasmid.

Since a wide variety of sanitizer reagents have been applied in food-processing environments in order to control and prevent biofilms, concerns have been raised over the inappropriate use of these reagents that might result in the emergence of sanitizer tolerant isolates due to adaptive response or even strains with cross-resistance to clinical antibiotics. A previous study showed that repeated exposure of *E. coli* strains to gradually increasing concentrations of QAC could lead to reduced susceptibility to the sanitizer and cross-resistance to phenicol compounds [111]. High tolerance was also observed in *Salmonella enterica* strains after exposure to an active biocidal compound [111]. Furthermore, prolonged exposure of *Salmonella* strains to sanitizers at sub-lethal concentrations could lead to selection of strains with reduced antibiotic susceptibility or even resistance to multiple antibiotics [106], in part due to the fact that such exposure might potentially select for strains with over-expressed efflux pumps. Interestingly, *Salmonella* serovar-related variation of cross-resistance between sanitizers and antimicrobial agents was reported previously [112,113]. On the other hand, available results have shown that bacterial antibiotic resistant strains were not necessary to be more tolerant to sanitizers than their antibiotic-sensitive counterparts [114]. In the future, the effect of sanitizer sub-lethal exposure on bacterial sanitizer tolerance or antibiotic/sanitizer cross-resistance requires further investigation.

## 6. Biofilm Community and Genetic Element Exchange

Bacterial coexistence in natural environment could lead to the development of mixed biofilms that constitute a reservoir to facility exchanges of genetic materials within the biofilm community via physical contact. Higher levels of horizontal gene transfer promoted within the biofilm community have been demonstrated in laboratories [115,116], and enhanced biofilm formation as a result of genetic material transfer has been observed as well. For instance, conjugative transfer of the F-like plasmid R1drd19 in *E. coli* strains [117] and transfer of plasmid RP4 between *Pseudomonas* species [118] were both shown to occur at significantly higher frequencies in biofilms. Studies [119,120] using *E. coli* systems also demonstrated horizontal transfer of non-conjugative plasmids and genetic elements. On the other hand, transmission of conjugative plasmids R1drd19 [121] and pMAS2027/pOLA52 [122] could induce greater biofilm development in *E*. *coli* cultures through pili and type 3 fimbriae synthesis, respectively. More importantly, it has been shown that the *E. coli* plasmid pOLA52 could retain its ability to induce biofilm development after it was transferred to other bacterial species, including *Salmonella* Typhimurium strains [123]. Transformation of non-conjugative plasmids pET28 and pUC8 could also increase biofilm cell growth in *E. coli* cultures [124].

In addition to facilitate plasmid transfer, biofilm development could also affect plasmid copy-number control, which has been investigated with plasmids pBR322 and pCF10. The copy-numbers of the two plasmids were both increased in biofilms compared to the planktonic cells in *E. coli* [125] and *E. faecalis* [126] cultures, respectively. The higher copy number of the plasmid pBR322, which encoded antibiotic resistance genes against ampicillin and tetracycline, was also correlated with stronger antibiotic resistance phenotype, as the presence of sub-lethal concentrations of the antibiotic increased the copy number of the plasmid [125]. A recent report [107] showed that compared to the diversity control panel strains, HEP *E. coli* O157:H7 strains overall retained significantly higher copy number of the pO157 plasmid, and a positive correlation was observed among the high plasmid copy number, strong biofilm forming ability, low sanitizer susceptibility, and high survival/recovery capability of the biofilm cells after sanitization. Since the highly conserved pO157 plasmid has been associated with biofilm formation and bacterial optimal survival/persistence in the environment [108,109,110], the high copy number of the pO157 plasmid might therefore constitute the genetic basis for the strong biofilm forming ability and high sanitizer tolerance of these HEP *E. coli* O157:H7 strains that pose stronger survival capability and higher potential of causing contamination at the meat plants.

Biofilm promoted horizontal gene transfer that might potentially lead to dissemination of antibiotic resistance determinants is another concern to food safety and public health. Increased transfer of multidrug resistance plasmids has been shown in *E. coli* [127,128] and *Staphylococcus aureus* biofilms [115]. Resistance against oxyimino-cephalosporins encoded by the bla CTX-M genes was investigated in *E. coli*, *K. pneumoniae,* and *E. cloacae*, and results also showed that the transfer frequency of the bla CTX-M genes was higher in strains at their biofilm stage than those at planktonic state [128]. In addition, it was reported that the natural blaNDM-1 plasmids could be successfully transferred from *E. coli* trans-conjugants to strong biofilm formers of *P. aeruginosa* and *A. baumannii* in dual-species biofilms, demonstrating the potential spread of the blaNDM-1 carbapenemase gene conferring resistance to carbapenem antibiotics within the multispecies biofilm community [129].

It is worthy to note that experiments performed under laboratory settings and longitudinal studies conducted at real commercial/industry environment involving animal population movements and antimicrobial usage over time may provide different observations. For instance, transfer of bla CMY-2-carrying plasmids conferring cephalosporins resistance was observed from *E. coli* donor strains to *Serratia marcescens* strains in biofilms, and the recipient strains with the resistant phenotype as a result of bla CMY-2 acquisition further acted as secondary plasmid donors [127]. However recently, a 24-month longitudinal study [130] reported that the presence of third-generation cephalosporins (3GC)-resistant *Salmonella* in commercial cattle feed yards was driven by the persistent pathogen subtypes instead of actively acquiring and maintaining the bla genes from the more frequently isolated 3GC-resistant *E. coli*, which has been suggested as the reservoir of 3GC resistance. Such observation contradicts the traditional reservoir theory, which was based on the isolation of *Salmonella* and *E. coli* harboring the IncA/C2 plasmids with similar genetic structures, including the bla CMY-2 gene, that may facilitate horizontal gene transfer between the two bacterial species. These various findings brought additional dimension to biofilm research concerning genetic material exchange under real life conditions because critical information, such as bacterial species, plasmid replicon profile, presence/location of antibiotic resistant genes, as well as evolutionary relationships, should be all taken into consideration when investigating biofilm related dissemination and persistence of genetic elements.

## 7. Conclusions

In the food-processing environment, the coexistence of multiple bacterial species profoundly affects biofilm structure, composition, and, more importantly, the tolerance levels of the biofilm cells to sanitizers and other antimicrobial interventions. Understanding how foodborne pathogens are protected and released into the food-processing environment from biofilms will lead to new knowledge on sanitizer tolerance and recurrent contamination. Studies related to foodborne pathogen or microbial interventions on biofilms are mostly designed on single-species biofilms or planktonic cells in axenic settings, overlooking the fact that in most environments, microorganisms coexist. Mixed-species biofilms are more tolerant to sanitizers than single-species biofilms or their planktonic equivalents. Hence, there is a need to explore how multispecies biofilms help in protecting the foodborne pathogen from common sanitizers and disseminate biofilm cells from hotspots and contaminated food products. This knowledge will help in designing microbial interventions to mitigate foodborne pathogens in the processing environment. As the global need for safe, high-quality, and nutritious food increases, it is vital to study foodborne pathogen behavior in a multi-partite interaction and engineer new interventions that safeguard food from contamination with pathogens.

## Data Availability

Not applicable.

## References

[1] Williams P. (2007). Quorum sensing, communication and cross-kingdom signalling in the bacterial world. Microbiology.

[2] Roder H.L., Raghupathi P.K., Herschend J., Brejnrod A., Knochel S., Sorensen S.J., Burmolle M. (2015). Interspecies interactions result in enhanced biofilm formation by co-cultures of bacteria isolated from a food processing environment. Food Microbiol..

[3] Frey-Klett P., Burlinson P., Deveau A., Barret M., Tarkka M., Sarniguet A. (2011). Bacterial-fungal interactions: Hyphens between agricultural, clinical, environmental, and food microbiologists. Microbiol. Mol. Biol. Rev..

[4] Abram F. (2015). Systems-based approaches to unravel multi-species microbial community functioning. Comput. Struct. Biotechnol. J..

[5] Joshi R.V., Gunawan C., Mann R. (2021). We Are One: Multispecies Metabolism of a Biofilm Consortium and Their Treatment Strategies. Front. Microbiol..

[6] McNeilly O., Mann R., Hamidian M., Gunawan C. (2021). Emerging Concern for Silver Nanoparticle Resistance in Acinetobacter baumannii and Other Bacteria. Front. Microbiol..

[7] Muller E.E.L., Faust K., Widder S., Herold M., Martínez Arbas S., Wilmes P. (2018). Using metabolic networks to resolve ecological properties of microbiomes. Curr. Opin. Syst. Biol..

[8] Yang J., Barrila J., Mark Ott C., King O., Bruce R., McLean R.J.C., Nickerson C.A. (2021). Longitudinal characterization of multispecies microbial populations recovered from spaceflight potable water. NPJ Biofilms Microbiomes.

[9] Donlan M.R. (2002). Biofilms: Microbial life on surfaces. Emerg. Infect. Dis..

[10] Oliveira N.M., Martinez-Garcia E., Xavier J., Durham W.M., Kolter R., Kim W., Foster K.R. (2015). Biofilm Formation As a Response to Ecological Competition. PLoS Biol..

[11] Burmolle M., Ren D., Bjarnsholt T., Sorensen S.J. (2014). Interactions in multispecies biofilms: Do they actually matter?. Trends Microbiol..

[12] Jefferson K.K. (2004). What drives bacteria to produce a biofilm?. FEMS Microbiol. Lett..

[13] Federle M.J., Bassler B.L. (2003). Interspecies communication in bacteria. J. Clin. Investig..

[14] Hughes D.T., Sperandio V. (2008). Inter-kingdom signalling: Communication between bacteria and their hosts. Nat. Rev. Microbiol..

[15] Zammuto V., Rizzo M.G., Spanò A., Genovese G., Morabito M., Spagnuolo D., Capparucci F., Gervasi C., Smeriglio A., Trombetta D. (2022). In vitro evaluation of antibiofilm activity of crude extracts from macroalgae against pathogens relevant in aquaculture. Aquaculture.

[16] Srey S., Jahid I.K., Ha S.-D. (2013). Biofilm formation in food industries: A food safety concern. Food Control.

[17] Van Houdt R., Michiels C.W. (2010). Biofilm formation and the food industry, a focus on the bacterial outer surface. J. Appl. Microbiol..

[18] Shi X., Zhu X. (2009). Biofilm formation and food safety in food industries. Trends Food Sci. Technol..

[19] Kumar C.G., Anand S.K. (1998). Significane of microbial biofilms in food industry: A review. Int. J. Food Microbiol..

[20] Scallan E., Hoekstra R.M., Angulo F.J., Tauxe R.V., Widdowson M.A., Roy S.L., Jones J.L., Griffin P.M. (2011). Foodborne illness acquired in the United States—Major pathogens. Emerg. Infect. Dis..

[21] CDC (2015). Surveillance for Foodborne Diseases Outbreaks United States, 2015.

[22] NIoH (NIH) (2002). Research on Microbial Biofilms.

[23] Bridier A., Sanchez-Vizuete P., Guilbaud M., Piard J.C., Naitali M., Briandet R. (2015). Biofilm-associated persistence of food-borne pathogens. Food Microbiol..

[24] Garrett T.R., Bhakoo M., Zhang Z. (2008). Bacterial adhesion and biofilms on surfaces. Prog. Nat. Sci..

[25] Hermansson M. (1999). The DLVO theory in microbial adhesion. Colloids Surf. B Biointerfaces.

[26] Jacob N.I., Israelachvili N. (1992). Intermolecular and Surface Forces.

[27] Wilson L.G., Everett L.G., Cullen S.J. (2018). Handbook of Vadose Zone Characterization & Monitoring.

[28] Chang Y.-I., Chang P.-K. (2002). The role of hydration force on the stability of the suspension of *Saccharomyces cerevisiae*—Application of the extended DLVO theory. Colloids Surf. A Physicochem. Eng. Asp..

[29] Ryan R.P., Dow J.M. (2008). Diffusible signals and interspecies communication in bacteria. Microbiology.

[30] Gracia-Gonzalo D., Pagán R. (2015). Influence of environmental factors on bacterial biofilms formation in the food industry: A review. J. Postdr. Res..

[31] de Jesus Pimentel-Filho N., de Freitas Martins M.C., Nogueira G.B., Mantovani H.C., Vanetti M.C.D. (2014). Bovicin HC5 and nisin reduce *Staphylococcus aureus* adhesion to polystyrene and change the hydrophobicity profile and Gibbs free energy of adhesion. Int. J. Food Microbiol..

[32] Yang L., Liu Y., Wu H., Hóiby N., Molin S., Song Z.J. (2011). Current understanding of multi-species biofilms. Int. J. Oral Sci..

[33] Madsen J.S., Røder H.L., Russel J., Sørensen H., Burmølle M., Sørensen S.J. (2016). Coexistence facilitates interspecific biofilm formation in complex microbial communities. Environ. Microbiol..

[34] Giaouris E., Heir E., Hebraud M., Chorianopoulos N., Langsrud S., Moretro T., Habimana O., Desvaux M., Renier S., Nychas G.J. (2014). Attachment and biofilm formation by foodborne bacteria in meat processing environments: Causes, implications, role of bacterial interactions and control by alternative novel methods. Meat Sci..

[35] Kuchma S.L., Connolly J.P., O’Toole G.A. (2005). A three-component regulatory system regulates biofilm maturation and type III secretion in Pseudomonas aeruginosa. J. Bacteriol..

[36] Stojicic S., Shen Y., Haapasalo M. (2013). Effect of the source of biofilm bacteria, level of biofilm maturation, and type of disinfecting agent on the susceptibility of biofilm bacteria to antibacterial agents. J. Endod..

[37] Davey M.E., Caiazza N.C., O’Toole G.A. (2003). Rhamnolipid Surfactant Production Affects Biofilm Architecture in Pseudomonas aeruginosa PAO1. J. Bacteriol..

[38] Rickard A.H., Gilbert P., High N.J., Kolenbrander P.E., Handley P.S. (2003). Bacterial coaggregation: An integral process in the development of multi-species biofilms. Trends Microbiol..

[39] Chua S.L., Liu Y., Yam J.K.H., Chen Y., Vejborg R.M., Tan B.G., Kjelleberg S., Tolker-Nielsen T., Givskov M., Yang L. (2014). Dispersed cells represent a distinct stage in the transition from bacterial biofilm to planktonic lifestyles. Nat. Commun..

[40] Wang R. (2019). Biofilms and Meat Safety: A Mini-Review. J. Food Prot..

[41] Chitlapilly Dass S., Anandappa A. (2017). Food Factory Genomics: Where Big Data Drives Quality and Food Safety. Food Prot. Trends.

[42] Steenackers H., Hermans K., Vanderleyden J., De Keersmaecker S.C.J. (2012). *Salmonella* biofilms: An overview on occurrence, structure, regulation and eradication. Food Res. Int..

[43] Vivian R.C. (2014). The Evaluation of Biofilm Formation and Sensitivity to Peracetic Acid of *Salmonella* spp. Isolated from Poultry Abattoir. Ph.D. Dissertation.

[44] Watnick P., Kolter R. (2000). Minireview: Biofilm, city of microbes. J. Bacteriol..

[45] Lewis K. (2001). Riddle of biofilm resistance. Antimicrob. Agents Chemother..

[46] Sofos J.N., Geornaras I. (2010). Overview of current meat hygiene and safety risks and summary of recent studies on biofilms, and control of *Escherichia coli* O157:H7 in nonintact, and *Listeria monocytogenes* in ready-to-eat, meat products. Meat Sci..

[47] Silagyi K., Kim S.-H., Martin Lo Y., Wei C.-I. (2009). Production of biofilm and quorum sensing by *Escherichia coli* O157:H7 and its transfer from contact surfaces to meat, poultry, ready-to-eat deli, and produce products. Food Microbiol..

[48] Cloak Orla M., Solow Barbara T., Briggs Connie E., Chen C.-Y., Fratamico Pina M. (2002). Quorum Sensing and Production of Autoinducer-2 in *Campylobacter* spp., *Escherichia coli* O157:H7, and *Salmonella enterica* Serovar Typhimurium in Foods. Appl. Environ. Microbiol..

[49] Choi Y., Lee Y., Lee S., Kim S., Lee J., Ha J., Oh H., Shin I.-S., Yoon Y. (2019). Microbial contamination including Vibrio cholerae in fishery auction markets in West Sea, South Korea. Fish. Aquat. Sci..

[50] Wieczorek K., Bomba A., Osek J. (2020). Whole-Genome Sequencing-Based Characterization of Listeria monocytogenes from Fish and Fish Production Environments in Poland. Int. J. Mol. Sci..

[51] Logan Savannah L., Thomas J., Yan J., Baker Ryan P., Shields Drew S., Xavier Joao B., Hammer Brian K., Parthasarathy R. (2018). The *Vibrio cholerae* type VI secretion system can modulate host intestinal mechanics to displace gut bacterial symbionts. Proc. Natl. Acad. Sci. USA.

[52] Chitlapilly Dass S., Abu-Ghannam N., Antony-Babu S., Cummins E.J. (2010). Ecology and molecular typing of L. monocytogenes in a processing plant for cold-smoked salmon in the Republic of Ireland. Food Res. Int..

[53] Chitlapilly Dass S., Cummins E.J., Abu-Ghannam N. (2011). Prevalence and Typing of Listeria Monocytogenes Strains in Retail Vacuum-Packed Cold-Smoked Salmon in the Republic of Ireland. J. Food Saf..

[54] Fox E.M., Solomon K., Moore J.E., Wall P.G., Fanning S. (2014). Phylogenetic profiles of in-house microflora in drains at a food production facility: Comparison and biocontrol implications of *Listeria*-Positive and -Negative bacterial populations. Appl. Environ. Microbiol..

[55] CDC (2015). Multistate Outbreak of Shiga Toxin-Producing Escherichia coli O157:H7 Infections Linked to Organic Spinach and Spring Mix Blend.

[56] CDC (2016). Multistate Outbreak of Shiga Toxin-Producing Escherichia coli O157 Infections Linked to Alfalfa Sprouts Produced by Jack & The Green Sprouts.

[57] CDC (2018). Multistate Outbreak of Shiga Toxin-Producing Escherichia coli O157:H7 Infections Linked to Leafy Greens (Final Update).

[58] Bogino P.C., Oliva M.D.I.M., Sorroche F.G., Giordano W. (2013). The role of bacterial biofilms and surface components in plant-bacterial associations. Int. J. Mol. Sci..

[59] Truong H.-N., Garmyn D., Gal L., Fournier C., Sevellec Y., Jeandroz S., Piveteau P. (2021). Plants as a realized niche for Listeria monocytogenes. MicrobiologyOpen.

[60] Holden N.J., Wright K.M., Marshall J., Holmes A., Schüller S., Bielaszewska M. (2021). Functional Analysis of Shiga Toxin-Producing *Escherichia coli* Biofilm Components in Plant Leaves. Shiga Toxin-Producing, E. coli: Methods and Protocols.

[61] Sun Y., Ma Y., Guan H., Liang H., Zhao X., Wang D. (2021). Adhesion mechanism and biofilm formation of *Escherichia coli* O157:H7 in infected cucumber (*Cucumis sativus* L.). Food Microbiol..

[62] Ávila-Quezada G., Sánchez E., Gardea-Béjar A.A., Acedo-Félix E. (2010). *Salmonella* spp. and *Escherichia coli*: Survival and growth in plant tissue. N. Z. J. Crop Hortic. Sci..

[63] Esteban-Cuesta I., Labrador M., Hunt K., Reese S., Fischer J., Schwaiger K., Gareis M. (2021). Phenotypic and Genetic Comparison of a Plant-Internalized and an Animal-Isolated *Salmonella* Choleraesuis Strain. Microorganisms.

[64] Grivokostopoulos N.C., Makariti I.P., Hilaj N., Apostolidou Z., Skandamis P.N. (2022). Internalization of *Salmonella* in leafy greens and the impact on acid tolerance. Appl. Environ. Microbiol..

[65] Arthur T.M., Bosilevac J.M., Brichta-Harhay D.M., Guerini M.N., Kalchayanand N., Shackelford S.D., Wheeler T.L., Koohmaraie M. (2007). Transportation and lairage environment effects on prevalence, numbers, and diversity of Escherichia coli O157:H7 on hides and carcasses of beef cattle at processing. J. Food Prot..

[66] Arthur T.M., Bosilevac J.M., Nou X., Shackelford S.D., Wheeler T.L., Koohmaraie M. (2007). Comparison of the molecular genotypes of Escherichia coli O157:H7 from the hides of beef cattle in different regions of North America. J. Food Prot..

[67] Rivera-Betancourt M., Shackelford S.D., Arthur T.M., Westmoreland K.E., Bellinger G., Rossman M., Reagan J.O., Koohmaraie M. (2004). Prevalence of Escherichia coli O157:H7, Listeria monocytogenes, and Salmonella in two geographically distant commercial beef processing plants in the United States. J. Food Prot..

[68] Stopforth J.D., Samelis J., Sofos J.N., Kendall P., Smith G.C. (2003). Influence of organic acid concentration on survival of Listeria monocytogenes and Escherichia coli 0157:H7 in beef carcass wash water and on model equipment surfaces. Food Microbiol..

[69] Stopforth J.D., Samelis J., Sofos J.N., Kendall P.A., Smith G.C. (2003). Influence of extended acid stressing in fresh beef decontamination runoff fluids on sanitizer resistance of acid-adapted Escherichia coli O157:H7 in biofilms. J. Food Prot..

[70] Wang R., Kalchayanand N., King D.A., Luedtke B.E., Bosilevac J.M., Arthur T.M. (2014). Biofilm formation and sanitizer resistance of *Escherichia coli* O157:H7 strains isolated from “high event period” meat contamination. J. Food Prot..

[71] Wang R., Luedtke B.E., Bosilevac J.M., Schmidt J.W., Kalchayanand N., Arthur T.M. (2016). *Escherichia coli* O157:H7 Strains Isolated from High-Event Period Beef Contamination Have Strong Biofilm-Forming Ability and Low Sanitizer Susceptibility, Which Are Associated with High pO157 Plasmid Copy Number. J. Food Prot..

[72] Wang R., Schmidt J.W., Harhay D.M., Bosilevac J.M., King D.A., Arthur T.M. (2017). Biofilm Formation, Antimicrobial Resistance, and Sanitizer Tolerance of *Salmonella enterica* Strains Isolated from Beef Trim. Foodborne Pathog. Dis..

[73] Stoodley P., Sauer K., Davies D.G., Costerton J.W. (2002). Biofilms as complex differentiated communities. Annu. Rev. Microbiol..

[74] da Silva Fernandes M., Coelho Alvares A.C., Martins Manoel J.G., Ramires Esper L.M., Kabuki D.Y., Kuaye A.Y. (2017). Formation of multi-species biofilms by Enterococcus faecium, Enterococcus faecalis, and Bacillus cereus isolated from ricotta processing and effectiveness of chemical sanitation procedures. Int. Dairy J..

[75] Wu H., Moser C., Wang H.Z., Hoiby N., Song Z.J. (2015). Strategies for combating bacterial biofilm infections. Int. J. Oral Sci..

[76] Wang H., Wang H., Xing T., Wu N., Xu X., Zhou G. (2016). Removal of *Salmonella* biofilm formed under meat processing environment by surfactant in combination with bio-enzyme. LWT Food Sci. Technol..

[77] Turnbull L., Toyofuku M., Hynen A.L., Kurosawa M., Pessi G., Petty N.K., Osvath S.R., Carcamo-Oyarce G., Gloag E.S., Shimoni R. (2016). Explosive cell lysis as a mechanism for the biogenesis of bacterial membrane vesicles and biofilms. Nat. Commun..

[78] Tan S.Y.-E., Chew S.C., Tan S.Y.-Y., Givskov M., Yang L. (2014). Emerging frontiers in detection and control of bacterial biofilms. Curr. Opin. Biotechnol..

[79] Ren D., Madsen J.S., Sorensen S.J., Burmolle M. (2015). High prevalence of biofilm synergy among bacterial soil isolates in cocultures indicates bacterial interspecific cooperation. ISME J..

[80] Afonso A.C., Gomes I.B., Saavedra M.J., Giaouris E., Simões L.C., Simões M. (2021). Bacterial coaggregation in aquatic systems. Water Res..

[81] Wu C., Chen Y.-W., Scheible M., Chang C., Wittchen M., Lee J.H., Luong T.T., Tiner B.L., Tauch A., Das A. (2021). Genetic and molecular determinants of polymicrobial interactions in *Fusobacterium nucleatum*. Proc. Natl. Acad. Sci. USA.

[82] Ledder R.G., Timperley A.S., Friswell M.K., Macfarlane S., McBain A.J. (2008). Coaggregation between and among human intestinal and oral bacteria. FEMS Microbiol. Ecol..

[83] Taga E.M., Bassler B.L. (2003). Chemical communication among bacteria. Proc. Natl. Acad. Sci. USA.

[84] Kuhl M., Rickelt L.F., Thar R. (2007). Combined imaging of bacteria and oxygen in biofilms. Appl. Environ. Microbiol..

[85] Karampatzakis A., Sankaran J., Kandaswamy K., Rice S.A., Cohen Y., Wohland T. (2017). Measurement of oxygen concentrations in bacterial biofilms using transient state monitoring by single plane illumination microscopy. Biomed. Phys. Eng. Express.

[86] Stewart P.S., Franklin M.J. (2008). Physiological heterogeneity in biofilms. Nat. Rev. Microbiol..

[87] Kurniawan A., Yamamoto T. (2019). Accumulation of NH^4+^ and NO_3_^−^ inside Biofilms of Natural Microbial Consortia: Implication on Nutrients Seasonal Dynamic in Aquatic Ecosystems. Int. J. Microbiol..

[88] Fuqua C., Parsek M.R., Greenberg E.P. (2001). Regulation of gene expression in cell to cell communication. Annu. Rev. Genet..

[89] Sztajer H., Szafranski S.P., Tomasch J., Reck M., Nimtz M., Rohde M., Wagner-Dobler I. (2014). Cross-feeding and interkingdom communication in dual-species biofilms of Streptococcus mutans and Candida albicans. ISME J..

[90] Tan C.H., Lee K.W.K., Burmolle M., Kjelleberg S., Rice S.A. (2017). All together now: Experimental multispecies biofilm model systems. Environ. Microbiol..

[91] Greenberg E.P. (2003). Bacterial communication and group behavior. J. Clin. Investig..

[92] Greenberg E.P. (2003). Bacterial communication: Tiny teamwork. Nature.

[93] Papenfort K., Bassler B.L. (2016). Quorum sensing signal-response systems in Gram-negative bacteria. Nat. Rev. Microbiol..

[94] Joint I., Tait K., Callow M.E., Callow J.A., Milton D., Williams P., Camara M. (2002). Cell-to-cell communication across the prokaryote-eukaryote boundary. Science.

[95] Brenner K., Karig D.K., Weiss R., Arnold F.H. (2007). Engineered bidirectional communication mediates a consensus in a microbial biofilm consortium. Proc. Natl. Acad. Sci. USA.

[96] Barathi S., Aruljothi K.N., Karthik C., Padikasan I.A., Ashokkumar V. (2022). Biofilm mediated decolorization and degradation of reactive red 170 dye by the bacterial consortium isolated from the dyeing industry wastewater sediments. Chemosphere.

[97] Lee K.W.K., Periasamy S., Mukherjee M., Xie C., Kjelleberg S., Rice S.A. (2014). Biofilm development and enhanced stress resistance of a model, mixed-species community biofilm. ISME J..

[98] Hall-Stoodley L., Costerton J.W., Stoodley P. (2004). Bacterial biofilms: From the natural environment to infectious diseases. Nat. Rev. Microbiol..

[99] Daniels R., Vanderleyden J., Michiels J. (2004). Quorum sensing and swarming migration in bacteria. FEMS Microbiol. Rev..

[100] Morris J.J., Lenski R.E., Zinser E.R. (2012). The Black Queen Hypothesis: Evolution of dependencies through adaptive gene loss. mBio.

[101] McMurry L.M., Oethinger M., Levy S.B. (1998). Triclosan targets lipid synthesis. Nature.

[102] Farr S.B., Kogoma T. (1991). Oxidative stress responses in *Escherichia coli* and *Salmonella typhimurium*. Microbiol. Rev..

[103] Seymour R.L., Mishra P.V., Khan M.A., Spector M.P. (1996). Essential roles of core starvation-stress response loci in carbon-starvation-inducible cross-resistance and hydrogen peroxide-inducible adaptive resistance to oxidative challenge in *Salmonella typhimurium*. Mol. Microbiol..

[104] Condell O., Iversen C., Cooney S., Power K.A., Walsh C., Burgess C., Fanning S. (2012). Efficacy of biocides used in the modern food industry to control *Salmonella enterica*, and links between biocide tolerance and resistance to clinically relevant antimicrobial compounds. Appl. Environ. Microbiol..

[105] Karatzas K.A.G., Webber M.A., Jorgensen F., Woodward M.J., Piddock L.J.V., Humphrey T.J. (2007). Prolonged treatment of *Salmonella enterica* serovar Typhimurium with commercial disinfectants selects for multiple antibiotic resistance, increased efflux and reduced invasiveness. J. Antimicrob. Chemother..

[106] White D.G., Goldman J.D., Demple B., Levy S.B. (1997). Role of the acrAB locus in organic solvent tolerance mediated by expression of marA, soxS, or robA in Escherichia coli. J. Bacteriol..

[107] Lim J.Y., Sheng H., Seo K.S., Park Y.H., Hovde C.J. (2007). Characterization of an Escherichia coli O157:H7 plasmid O157 deletion mutant and its survival and persistence in cattle. Appl. Environ. Microbiol..

[108] Lim J.Y., La H.J., Sheng H., Forney L.J., Hovde C.J. (2010). Influence of Plasmid pO157 on *Escherichia coli* O157:H7 Sakai Biofilm Formation. Appl. Environ. Microbiol..

[109] Lim J.Y., Hong J.B., Sheng H., Shringi S., Kaul R., Besser T.E., Hovde C.J. (2010). Phenotypic diversity of *Escherichia coli* O157:H7 strains associated with the plasmid O157. J. Microbiol..

[110] Soumet C., Fourreau E., Legrandois P., Maris P. (2012). Resistance to phenicol compounds following adaptation to quaternary ammonium compounds in *Escherichia coli*. Vet. Microbiol..

[111] Braoudaki M., Hilton A.C. (2004). Adaptive resistance to biocides in *Salmonella enterica* and *Escherichia coli* O157 and cross-resistance to antimicrobial agents. J. Clin. Microbiol..

[112] Futoma-Kołoch B., Książczyk M., Korzekwa K., Migdał I., Pawlak A., Jankowska M., Kędziora A., Dorotkiewicz-Jach A., Bugla-Płoskońska G. (2015). Selection and electrophoretic characterization of *Salmonella enterica* subsp. enterica biocide variants resistant to antibiotics. Pol. J. Vet. Sci..

[113] Karatzas K.A.G., Randall L.P., Webber M., Piddock L.J.V., Humphrey T.J., Woodward M.J., Coldham N.G. (2008). Phenotypic and Proteomic Characterization of Multiply Antibiotic-Resistant Variants of *Salmonella enterica* Serovar Typhimurium Selected Following Exposure to Disinfectants. Appl. Environ. Microbiol..

[114] Molin S., Tolker-Nielsen T. (2003). Gene transfer occurs with enhanced efficiency in biofilms and induces enhanced stabilisation of the biofilm structure. Curr. Opin. Biotechnol..

[115] Savage V.J., Chopra I., O’Neill A.J. (2013). Staphylococcus aureus biofilms promote horizontal transfer of antibiotic resistance. Antimicrob. Agents Chemother..

[116] Licht T.R., Christensen B.B., Krogfelt K.A., Molin S. (1999). Plasmid transfer in the animal intestine and other dynamic bacterial populations: The role of community structure and environment. Microbiology.

[117] Ehlers L.J., Bouwer E.J. (1999). Rp4 plasmid transfer among species of pseudomonas in a biofilm reactor. Water Sci. Technol..

[118] Tsen S.D., Fang S.S., Chen M.J., Chien J.Y., Lee C.C., Tsen D.H.L. (2002). Natural plasmid transformation in *Escherichia coli*. J. Biomed. Sci..

[119] Jeanson S., Floury J., Gagnaire V., Lortal S., Thierry A. (2015). Bacterial Colonies in Solid Media and Foods: A Review on Their Growth and Interactions with the Micro-Environment. Front. Microbiol..

[120] Yang X., Ma Q., Wood T.K. (2008). The R1 Conjugative Plasmid Increases *Escherichia coli* Biofilm Formation through an Envelope Stress Response. Appl. Environ. Microbiol..

[121] Ong C.-L.Y., Beatson S.A., McEwan A.G., Schembri M.A. (2009). Conjugative Plasmid Transfer and Adhesion Dynamics in an *Escherichia coli* Biofilm. Appl. Environ. Microbiol..

[122] Burmølle M., Bahl M.I., Jensen L.B., Sørensen S.J., Hansen L.H. (2008). Type 3 fimbriae, encoded by the conjugative plasmid pOLA52, enhance biofilm formation and transfer frequencies in Enterobacteriaceae strains. Microbiology.

[123] Teodósio J.S., Simões M., Mergulhão F.J. (2012). The influence of nonconjugative *Escherichia coli* plasmids on biofilm formation and resistance. J. Appl. Microbiol..

[124] May T., Ito A., Okabe S. (2009). Induction of multidrug resistance mechanism in Escherichia coli biofilms by interplay between tetracycline and ampicillin resistance genes. Antimicrob. Agents Chemother..

[125] Cook L., Chatterjee A., Barnes A., Yarwood J., Hu W.-S., Dunny G. (2011). Biofilm growth alters regulation of conjugation by a bacterial pheromone. Mol. Microbiol..

[126] Król J.E., Nguyen H.D., Rogers L.M., Beyenal H., Krone S.M., Top E.M. (2011). Increased transfer of a multidrug resistance plasmid in Escherichia coli biofilms at the air-liquid interface. Appl. Environ. Microbiol..

[127] Mo S.S., Sunde M., Ilag H.K., Langsrud S., Heir E. (2017). Transfer Potential of Plasmids Conferring Extended-Spectrum-Cephalosporin Resistance in *Escherichia coli* from Poultry. Appl. Environ. Microbiol..

[128] Maheshwari M., Ahmad I., Althubiani A.S. (2016). Multidrug resistance and transferability of *bla*_CTX-M_ among extended-spectrum β-lactamase-producing enteric bacteria in biofilm. J. Glob. Antimicrob. Resist..

[129] Tanner W.D., Atkinson R.M., Goel R.K., Toleman M.A., Benson L.S., Porucznik C.A., VanDerslice J.A. (2017). Horizontal transfer of the *bla*_NDM-1_ gene to *Pseudomonas aeruginosa* and *Acinetobacter baumannii* in biofilms. FEMS Microbiol. Lett..

[130] Schmidt J.W., Murray S.A., Dickey A.M., Wheeler T.L., Harhay D.M., Arthur T.M. (2022). Twenty-Four-Month Longitudinal Study Suggests Little to No Horizontal Gene Transfer In Situ between Third-Generation Cephalosporin-Resistant *Salmonella* and Third-Generation Cephalosporin-Resistant *Escherichia coli* in a Beef Cattle Feedyard. J. Food Prot..

